# Comprehensive identification of novel proteins and N-glycosylation sites in royal jelly

**DOI:** 10.1186/1471-2164-15-135

**Published:** 2014-02-16

**Authors:** Lan Zhang, Bin Han, Rongli Li, Xiaoshan Lu, Aiying Nie, Lihai Guo, Yu Fang, Mao Feng, Jianke Li

**Affiliations:** 1 Institute of Apicultural Research, Chinese Academy of Agricultural Science, Beijing 100093, China; 2 College of Bioengineering, Henan University of Technology, Zhengzhou 450001, China; 3 Bioengineering Department, Zhengzhou University, Zhengzhou 450001, China; 4 Thermo Fisher Scientific (China) Co., Ltd, Shanghai 200021, China; 5 Shanghai AB Sciex Analytical Instrument Trading Co., Ltd, Shanghai 200023, China

**Keywords:** Royal jelly, N-glycosylation, Hydrazide chemistry, Lectin affinity, Tandem mass spectrometry

## Abstract

**Background:**

Royal jelly (RJ) is a proteinaceous secretion produced from the hypopharyngeal and mandibular glands of nurse bees. It plays vital roles in honeybee biology and in the improvement of human health. However, some proteins remain unknown in RJ, and mapping N-glycosylation modification sites on RJ proteins demands further investigation. We used two different liquid chromatography-tandem mass spectrometry techniques, complementary N-glycopeptide enrichment strategies, and bioinformatic approaches to gain a better understanding of novel and glycosylated proteins in RJ.

**Results:**

A total of 25 N-glycosylated proteins, carrying 53 N-glycosylation sites, were identified in RJ proteins, of which 42 N-linked glycosylation sites were mapped as novel on RJ proteins. Most of the glycosylated proteins were related to metabolic activities and health improvement. The 13 newly identified proteins were also mainly associated with metabolic processes and health improvement activities.

**Conclusion:**

Our in-depth, large-scale mapping of novel glycosylation sites represents a crucial step toward systematically revealing the functionality of N-glycosylated RJ proteins, and is potentially useful for producing a protein with desirable pharmacokinetic and biological activity using a genetic engineering approach. The newly-identified proteins significantly extend the proteome coverage of RJ. These findings contribute vital and new knowledge to our understanding of the innate biochemical nature of RJ at both the proteome and glycoproteome levels.

## Background

Royal jelly (RJ) is a proteinaceous secretion derived from the hypopharyngeal and mandibular glands of young worker bees [1,2]. It is the sole food fed to the queen throughout her lifetime, and is also fed to all young larvae for the first three days after hatching [2]. RJ possesses various biological attributes beneficial for human health, such as antioxidant activities [3], antibacterial effects [4], enhancement of immune activity [5], and antitumor effects [6]. Protein accounts for >50% of RJ by dry weight [2]. It has been reported that nine members of major royal jelly proteins (MRJPs, MRJP1-9) [7,8] account for 80-90% of the total protein in RJ [9]. Other proteins, such as alpha-glucosidase, glucose oxidase, and alpha-amylase have also been detected in RJ [1,10-12]. Although several studies have indicated that the proteins in RJ have undergone glycosylation modification [12-16], we do not yet know the types or site assignments of this glycoprotein. With the development of new technologies in protein separation and identification, dozens of novel proteins have been recently identified in RJ by our group and by others [1,11,16,17]. Advances in resolution and sensitivity (double high) of liquid chromatography-tandem mass spectrometry (LC-MS/MS) have made it a powerful platform. These advances have made it possible to profile the proteome of RJ more deeply, while allowing for system-level mapping of glycosylation sites of RJ proteins.

Asparagine-linked (N-linked) protein glycosylation is the most abundant of all posttranslational modifications in eukaryotes, with nearly 70% of all eukaryotic proteins predicted to be N-glycoproteins [18]. N-linked glycosylation is an enzymatically catalyzed process that occurs in the endoplasmic reticulum (ER). It involves the assembly of glycans on a lipid carrier in the ER membrane, followed by a transfer to specific asparagine residues of target polypeptides [19]. The attachment of N-glycans to a peptide backbone has been reported to assist in protein folding, stability, solubility, oligomerization, quality control, sorting, and transport [20,21]. Glycoproteins mediate many important biological processes by their involvement in cell adhesion, cell differentiation, cell growth, and immunity [22,23].

To identify N-glycosylated peptides from the more abundant non-glycosylated peptides in complex biological samples, specific enrichment methods, such as lectin affinity [24] or hydrazide chemistry [25], are required before they are subjected to double high LC-MS/MS analysis. Since a consensus sequence motif of N-X-S/T exists in N-glycosylation [20,21] (N = asparagine, X = any amino acid except proline, S/T = serine or threonine), the digested asparagine residue in N-X-S/T resulting from deglycosylation of the enzyme (Peptide N Glycosidase, PNGase F, commonly used) usually increases the mass by 0.98 Da. This basic scientific evidence is used to locate the N-glycosylation sites on a protein [26]. For more exact mapping of N-glycosylation sites, deglycosylation is usually done by introduction of ^18^O-water (H_2_^18^O), which increases a mass shift in the MS spectra of 2.99 Da, thus adding confidence to the site assignment [27].

It is well-known that mapping residue-specific glycosylation sites is the first step towards a detailed and functional understanding of proteins [20]. However, information on N-glycosylation site assignment in RJ proteins is still very limited, thus demanding a powerful glycoproteomics approach to large-scale comprehensive mapping N-glycosylated sites in RJ proteins. Until now, RJ proteins have been documented to contain a series of glycoproteins [12,14,15], and are potentially glycosylated by a gel stain [28]. Only MRJP 2 is reported to carry two N-glycosylated sites attached a high-mannose structure and complex type antennary structures [16].

In an effort to identify hidden proteins and to map the N-linked glycosylation sites in RJ, two different double high LC-MS/MS systems, Q-Exactive coupled to Easy-nLC 1000 (orbitrap-based MS) and Triple TOF 5600 coupled with an Eksigent nLC (triple TOF-based MS), as well as complementary glycopeptide enrichment protocols (hydrazide and lectin), were employed. Overall, 25 N-glycosylated proteins carrying 53 N-glycosylation sites were confidently identified, of which novel 42 N-linked glycosylation sites were mapped in RJ proteins, and 13 novel proteins were identified in RJ.

## Results

### Identified novel royal jelly proteins

To expand the number of known proteins in the RJ proteome, RJ proteins were extracted and digested with in-solution methods and analyzed with double high LC-MS/MS (orbitrap-based MS). A total of 42 nonredundant proteins were confidentially identified, of which 13 proteins were novel (Table 1 and Additional file 1: Table S1).

**Table 1:** Identification of proteins in royal jelly

**Classification**	**Accession**	**-10lgP**	**Coverage (%)**	**Matches**	**Unique**	**Mass(Da)**	**Protein name**	**SignalP**	**PSORT**
**YELLOW/MRJP family**	gi|58585098	566.72	96	742	166	48886	Major royal jelly protein 1 precursor	-	-
	gi|58585108	527.66	93	437	100	51074	Major royal jelly protein 2 precursor	-	-
	gi|58585142	487.03	88	354	12	61662	Major royal jelly protein 3 precursor	-	-
	gi|284182838	357.38	89	236	15	53015	Major royal jelly protein 4	-	-
	gi|284812514	386.92	71	182	6	70182	Major royal jelly protein 5	-	-
	gi|58585188	282.69	58	57	7	49786	Major royal jelly protein 6 precursor	-	-
	gi|62198227	422.37	84	182	70	50541	Major royal jelly protein 7 precursor	-	-
	gi|58585070	131.13	15	3	3	46956	Major royal jelly protein 8 precursor	-	-
	gi|67010041	221.7	45	26	10	48688	Major royal jelly protein 9 precursor	-	-
	gi|148277624	146.46	19	6	6	48235	Yellow-e3 precursor*	√	#
**Metabolic activity**	gi|328787887	299.74	26	40	37	188194	Lysosomal alpha-mannosidase	-	-
	gi|89885579	290.12	50	33	3	65565	Alpha-glucosidase	-	-
	gi|58585144	227.31	43	7	3	55947	Alpha-amylase precursor	-	-
	gi|66564326	185.29	37	10	6	52947	Plasma glutamate carboxypeptidase isoform 1	-	-
	gi|328778095	116.42	10	4	4	56432	Lysosomal Pro-X carboxypeptidase*	√	#
	gi|66560290	102.39	10	3	3	42222	Lysosomal aspartic protease*	√	#
	gi|328782027	93.88	6	4	4	88720	Membrane metallo-endopeptidase 1*	√	#
	gi|48118838	276.66	56	32	31	58571	Glucosylceramidase	-	-
	gi|66524161	196.85	59	15	14	25186	Ferritin heavy chain	-	-
	gi|328779534	220.38	10	23	20	79344	Hypothetical protein LOC552041	-	-
	gi|328780642	167.48	16	9	8	64654	Matrix metalloproteinase 14*	√	#
	gi|328784061	154.24	27	6	5	34132	Pancreatic triacylglycerol lipase*	√	#
Health promotion activity	gi|58585090	389.52	76	137	77	67938	Glucose oxidase	-	-
	gi|166795901	259.92	93	46	14	21348	Apolipophorin-III protein precursor	-	-
	gi|328782084	240.41	41	7	4	59502	Antithrombin-III	-	-
	gi|60115688	226.26	71	39	5	24819	Icarapin precursor	-	-
	gi|254910938	214.17	75	14	4	10717	Defensin-1 preproprotein	-	-
	gi|187281543	150.07	16	11	10	87937	Venom dipeptidyl Peptidase 4 precursor*	√	#
	gi|110755367	142.44	13	7	7	75706	Toll-like receptor 13 isoform 1	-	-
	gi|48101366	138.63	15	5	5	44639	Venom serine protease 34*	√	#
	gi|254548157	102.39	37	3	2	12611	Hymenoptaecin precursor*	√	#
	gi|328790726	243.78	70	28	19	42665	Venom acid phosphatase Acph 1	-	-
	gi|66507455	188.31	27	11	11	39483	Venom protease*	√	#
	gi|328792524	89.27	9	5	2	90763	Hypothetical protein LOC408570*	√	#
	gi|110758964	221.47	83	17	4	10161	Regucalcin	-	-
	gi|66565246	83.06	16	3	2	17081	Lysozyme isoform 1*	√	#
Developmental process	gi|66514614	165.33	19	8	4	48741	Idgf4	-	-
	gi|110766389	139.29	22	5	5	30201	Protein takeout	-	-
	gi|66521538	101.17	11	2	2	33735	Protein CREG1*	√	#
	gi|94158822	86.71	22	2	2	15201	Odorant binding protein 14 precursor	-	-
Unknown	gi|48094573	292.99	64	45	32	19434	Hypothetical protein LOC408608	-	-
	gi|110763647	109.47	27	5	4	18478	Hypothetical protein LOC726323	-	-

Note: All of the identified proteins are of *Apis mellifera* origin. Accession is the unique number given to mark the entry of a protein in the database of *Apis* (downloaded April 2012, version 4.5 of the honeybee genome) using in-house PEAKS software (version 6.0, Bioinformatics Solutions Inc.). “-10logP” is the score calculated by PEAKS software. Sequence coverage is the ratio of the number of amino acids in every peptide that matches with the mass spectrum divided by the total number of amino acids in the protein sequence. Matches are the number of experiment fragmentation spectra paired to a theoretical segment of protein. The number of unique peptides refers to the peptide sequences that are unique to an individual parent protein sequence. SignalP refers to the result researched with SignalP 4.1. PSORT refers to the result researched with PSORT II. “*” indicates the protein identified as novel in royal jelly. “√” indicates the protein identified with signal peptide by SignalP 4.1. “#” indicates the protein identified as extracellular by PSORT II. “-” indicates the protein did not be researched with SignalP 4.1 or PSORT II.

The 42 identified proteins in RJ were classified on the basis of their biological process and molecular function and annotated by gene ontology. In the YELLOW/MRJP family, a new protein, yellow-e3 precursor, was identified. Of the 12 proteins related to metabolic processes, five novel proteins were identified: lysosomal pro-X carboxypeptidase, lysosomal aspartic protease, membrane metallo-endopeptidase 1, matrix metalloproteinase 14, and pancreatic triacylglycerol lipase. Among the 14 proteins associated with health improvement, six were reported here for the first time: venom dipeptidyl peptidase 4 precursor, venom serine protease 34, hymenoptaecin precursor, venom protease, hypothetical protein LOC408570, lysozyme isoform 1. One of the four proteins involved in development processes was novel, protein CREG 1 (Table 1 and Additional file 1: Table S1). Interestingly, the majority of the newly-identified proteins were related both to metabolic processes (accounting for 38.5% of all novel proteins) and health promotion activities (46.2% of all novel proteins).

### Mapping N-glycosylated sites

**Figure 1:**
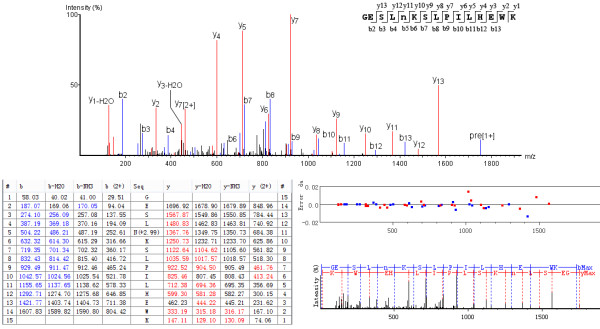
**Representative spectra of N-glycosylated peptide in royal jelly proteins.** The tandem mass spectrum of the N-glycosylated site is identified in peptide GESLN(+2.99)KSLPILHEWK using ^18^O-water labeling.

To attain a comprehensive map of N-linked glycosylation sites in RJ, RJ proteins were extracted and enriched by two different enrichment methods (hydrazide and lectin), after which the N-glycosylation peptides were analyzed by two different double high LC-MS/MS (orbitrap-based MS and triple TOF-based MS). The introduction of ^18^O-water in the process of PNGase F digestion added to confidence to the identification of N-glycopeptides. An example spectrum of N-glycopeptide is shown in Figure 1 (for all other spectra see Additional file 2: Figure S1). Overall, 25 N-glycoproteins carrying 53 unique N-linked glycosylation sites represented 60% of the total identified proteins in RJ. Among the 53 identified N-linked glycosylation sites, 42 were confidentially mapped in RJ proteins for the first time (Table 2).

**Table 2:** Identification of glycosylated proteins, peptides and their glycosylation sites in royal jelly proteins

**Accession**	**Glycoprotein and glycopeptide**	**-10lgP**	**Mass(Da)**	**Charge**	**No. of spectra**	**Amino acid residue no.**	**Glycosylation site (N-type)**	**Enrichment method**			
								**Lectin**		**Hydrazide**	
								**Orbitrap**	**Triple TOF**	**Orbitrap**	**Triple TOF**
gi|58585098	**Major royal jelly protein 1 precursor**	442.09	48886								
	R.GESLN(+2.99)KSLPILHEWK.F^b^	57.82	585.32	3	8	24-38	28	√		√	
gi|58585108	**Major royal jelly protein 2 precursor**	398.06	51074								
	K.TSNHLKQIEIPHDIAVN(+2.99)ATTGK.G^a^	67.92	797.42	3	15	162-183	178	√	√	√	√
	K.IAIDKFDRLWVLDSGLVN(+2.99)R.T^a^	57.57	1117.1	2	9	128-146	145	√	√	√	√
gi|284182838	**Major royal jelly protein 4**	298.73	53015								
	R.KN(+2.99)LTNTLNVIHEWK.Y^b^	57.71	856.97	2	5	30-41	31	√		√	
	K.M(+15.99)SNQQEN(+2.99)LTLKEVDNK.V^b^	47.23	637.31	3	2	236-251	242	√		√	
gi|58585188	**Major royal jelly protein 6 precursor**	203.5	49786								
	K.NYPFDVDQWHN(+2.99)K.T^d^	47.63	783.35	2	4	68-79	78	√		√	
	K.LLAFDLN(+2.99)TSKLLK.Q^d^	47	739.94	2	2	158-170	164		√		
gi|62198227	**Major royal jelly protein 7 precursor**	312.92	50541								
	K.QVDIPHEIAVN(+2.99)TTTEQGR.L^d^	54.49	1006	2	11	168-185	178	√	√	√	√
	R.LWVLDSGLVN(+2.99)NTQPM(+15.99)C(+57.02)FPK.L^d^	45.55	1112	2	8	136-154	145	√	√	√	
	K.NGILFFGLVN(+2.99)NTAVGC(+57.02)WNEHQ(+0.98)TLQ(+0.98)R.E^d^	57.66	1447.2	2	2	312-316	321			√	
gi|67010041	**Major royal jelly protein 9 precursor**	107.71	48688								
	K.IPHDIAIN(+2.99)STTGK.R^d^	40.14	685.37	2	2	170-182	177	√			
gi|148277624	**Yellow-e3 precursor**	89	48235								
	K.YM(+15.99)SGTLNSN(+2.99)ETNFR.I^e^	50.32	826.86	2	2	384-397	392			√	
gi|328787887	**Lysosomal alpha-mannosidase**	165.89	188194								
	R.LTQSFHYYEGM(+15.99)EGNNM(+15.99)EFKN(+2.99)R.S^d^	53.93	877.38	3	4	538-558	557	√		√	
	R.LLKDDAFGVGEALN(+2.99)ESAYGEGLVVR.G^d^	50.85	1313.2	2	2	722-746	735	√			
gi|89885579	**Alpha-glucosidase**	193.65	65565								
	K.N(+2.99)VSRDSN(+2.99)SSDFKK.L^b^	39.74	497.24	3	2	313-325	313 319	√			
	K.HM(+15.99)LIEAYTN(+2.99)LSM(+15.99)TM(+15.99)K.Y^b^	38.65	917.42	2	2	282-296	290		√		
gi|66564326	**Plasma glutamate carboxypeptidase isoform 1**	178.08	52947								
	K.ESADYGLENVHGEN(+2.99)VTVPFWVR.G^d^	63.89	1261.1	2	2	99-120	112	√			
	R.SVTPYSLYTPHTGHQSYGEN(+2.99)VTK.I^d^	55.98	643.06	4	5	212-234	231	√		√	
	R.IM(+15.99)TLLSPM(+15.99)GN(+2.99)LTVR.S^d^	42.7	790.92	2	3	394-407	403	√	√		
	R.AIM(+15.99)NEALN(+2.99)GSFK.G^d^	34.47	657.33	2	2	54-65	61		√		
gi|328778095	**Lysosomal Pro-X carboxypeptidase**	71.61	56432								
	R.YYGESM(+15.99)PYNN(+2.99)K.S^e^	35.71	692.79	2	1	124-134	133			√	
gi|66560290	**Lysosomal aspartic protease**	62.1	42222								
	K.N(+2.99)GTDFAIR.Y^e^	35.21	448.72	2	1	120-127	120	√			
gi|328782027	**Membrane metallo-endopeptidase 1**	89.03	88720								
	K.HNPIPDN(+.98)KVEWSEDEIKAN(+2.99)K.T^e^	50.01	592.55	4	2	92-111	110	√			
	K.WYDN(+.98)SGVN(+2.99)TSTAK.I^e^	37.35	723.83	2	1	311-323	318	√			
	R.IVNTN(+2.99)DTETR.L^e^	36.04	583.28	2	1	31-40	35	√			
gi|48118838	**Glucosylceramidase**	142.8	58571								
	K.QFDNN(+2.99)ITYLKEEHYETYVNYLIK.F^d^	54.42	735.86	4	3	211-233	215	√		√	
	K.N(+2.99)FSLAPEDYNYK.I^d^	46.71	732.33	2	2	171-182	171	√			
	K.TQANWIANYFGPILASSPFN(+2.99)K.T^d^	30.3	781.41	3	1	273-293	292		√		
	R.M(+15.99)N(+2.99)VSEVKFDR.C^d^	40.85	622.3	2	3	72-81	73	√	√	√	
gi|58585090	**Glucose oxidase**	277.69	67938								
	R.SNLHVIVN(+2.99)ATVTK.V^d^	54.93	699.9	2	8	277-289	284	√	√	√	√
	K.LVN(+2.99)TTVM(+15.99)RDLGVEFQK.I^d^	52.05	934.99	2	7	499-514	501	√	√	√	√
	R.WVQQGAFGWSWDEVM(+15.99)PYYLKSEN(+2.99)NTELSR.V^d^	40.6	1180.9	3	4	172-200	194			√	√
	R.AFITPFEN(+2.99)R.S^d^	41.19	549.28	2	3	268-276	275			√	√
	K.YYTTN(+2.99)ESHACLSTGGSCYWPR.G^d^	41.83	800.34	3	2	126-146	130			√	
gi|166795901	**Apolipophorin-III-like protein precursor**	226.7	21348								
	K.DQSANFVNNIQDYIKN(+2.99)VTEEVK.T^d^	62.84	857.76	3	6	71-92	86	√	√	√	
gi|328782084	**Antithrombin-III**	97.9	59502								
	K.ISN(+2.99)DSAQNGERDSIYHLIER.L^d^	48.05	580.78	4	2	362-381	364	√			
gi|187281543	**Venom dipeptidyl peptidase 4 precursor**	80.93	87937								
	R.HLAFATFN(+2.99)DTNVR.D^c^	47.19	503.59	3	2	232-244	239	√			
	R.ANSFN(+2.99)GTWK.T^c^	39.89	514.24	2	2	64-72	68	√			
	K.YSWIDSN(+2.99)R.T^c^	34.35	522.24	2	2	625-632	631			√	
gi|110755367	**Toll-like receptor 13 isoform 1**	127.33	75706								
	R.M(+15.99)LEHLDLSN(+2.99)NSLSTVNR.R^d^	55.39	981.48	2	2	547-563	555	√			
	R.HLNTQFFHN(+2.99)TTNLNK.L^d^	54.76	611.31	3	2	166-180	174	√			
	K.LHTLEEGLFAN(+2.99)LTR.L^d^	50.96	539.62	3	2	432-445	442	√			
	R.LSEEAFKN(+2.99)ASK.L^d^	39.92	613.81	2	1	315-325	322	√			
gi|328790726	**Venom acid phosphatase Acph-1**	142.25	42665								
	K.M(+15.99)PSTINFYPNDPYIN(+2.99)YTYEPAGK.G^d^	57.47	1357.6	2	2	38-60	52	√			
gi|328792524	**Hypothetical protein LOC408570**	89.27	90763								
	R.WSLTPVNSN(+2.99)TTVVVK.Q^e^	43.11	824.45	2	2	539-553	547	√			
	R.QN(+2.99)YTDAPPAK.L^e^	41.13	554.27	2	2	590-599	591	√			
	R.IDPN(+2.99)SSFTQSNPIR.F^e^	38.14	789.89	2	2	284-297	287	√		√	
gi|66514614	**Idgf4**	135.8	48741								
	R.LKDLTIGVLPHVN(+2.99)STVYYDAR.L^d^	57.43	595.07	4	2	216-236	228	√			
gi|110766389	**Protein takeout**	90.9	30201								
	R.ALFSN(+2.99)ITVIGAGN(+2.99)YSLTK.S^d^	56.15	938	3	2	105-122	109 117	√		√	
gi|48094573	**Hypothetical protein LOC408608**	292.9	19434								
	K.GNLGTVN(+2.99)LTKVLKSVEDR.L^d^	53.12	641.34	4	5	61-78	67	√	√	√	√
gi|110763647	**Hypothetical protein LOC726323**	109.47	18478								
	R.IYDPITN(+2.99)TSK.M^d^	35.5	577.8	2	1	133-142	139			√	

Note: All of the identified proteins are from *Apis mellifera*. Accession is the unique number given to mark the entry of a protein in the database of *Apis* (downloaded April 2012, version 4.5 of the honeybee genome). “-10logP” is the score calculated by PEAKS software (version 6.0, Bioinformatics Solutions Inc.). Charge is the number of the carrying charge of the peptide. No. of spectra is the number of the spectrum of the peptide generated by mass spectrometry. Amino acid residue No. corresponds to the position of the N-terminal and C-terminal amino acid of the peptide in the protein sequence. Glycosylation site indicates the position of the N-glycosylated amino acids of the peptide in the protein sequence. Orbitrap refers the peptides analyzed by the Q-Exactive mass spectrometry (Thermo Fisher Scientific). Triple TOF refers the peptides analyzed by Triple TOF 5600 (AB SCIEX). Lectin denotes N-glycopeptides enriched by the lectin method. Hydrazide represents N-glycopeptides enriched by hydrazide chemistry. “√” indicates that peptide is identified by the corresponding enrichment method and mass spectrometer. “a” is the known site in the known protein. “b” is the potential site (bioinformatics has predicted these potential sites in UniProt Database (updated April 2013), and it is experimentally confirmed in this study) in the known protein. “c” is the potential site in the novel protein. “d” denotes the novel site in the known protein. “e” is the novel site in the novel protein.

In the YELLOW/MRJP family, seven proteins were identified as N-glycoproteins, glycosylated on 12 unique peptides, each carrying a single N-glycosylated site (Table 2). Of the proteins involved in metabolic processes, seven were N-glycosylated on 16 unique N-glycopeptides: all but on each contained a single N-glycosylation site and one unique N-glycopeptide carried two sites (Table 2). Of the proteins related to health improvement, seven were found N-glycosylated on 18 unique peptides, and each peptide had a single N-glycosylated site (Table 2). Of the two proteins implicated in the regulation of morphological development, IDGF 4 was N-glycosylated on one unique peptide with a single site, and N-glycosylated protein takeout had one unique peptide carrying two sites (Table 2). Finally, two identified N-glycoproteins with unknown functions each had one unique peptide harboring a single N-glycosylated site (Table 2).

**Figure 2:**
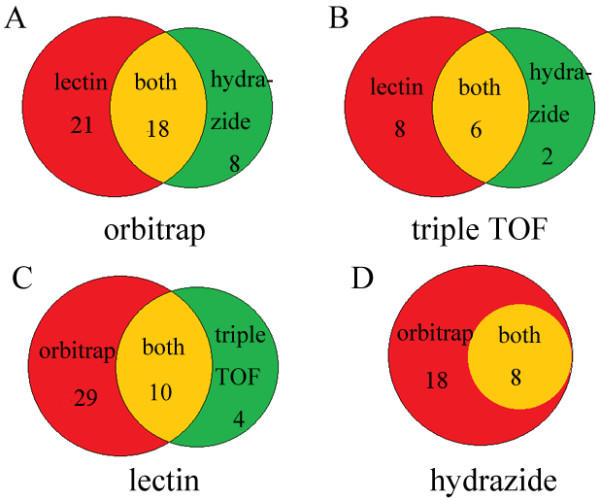
**Distribution of N-glycopeptides analyzed by different enriched methods and instruments of royal jelly proteins. A** is the distribution of N-glycopeptides enriched by lectin and hydrazide methods using mass spectrometry of Q-Exactive (orbitrap-based MS). 21 and eight are N-glycopeptides uniquely identified by the lectin and hydrazide enrichment, respectively, and 18 are N-glycopeptides identified by both enrichment methods using orbitrap-based MS. **B** is the distribution of N-glycopeptides enriched by lectin and hydrazide methods using mass spectrometry of triple TOF 5600 (triple TOF-based MS). Eight and two are N-glycopeptides specifically identified by the lectin and hydrazide enrichment protocols, respectively, and six are N-glycopeptides identified by both enrichment methods using triple TOF-based MS. **C** is the distribution of N-glycopeptides identified by the orbitrap-based MS and triple TOF-based MS using lectin enrichment method. 29 are N-glycopeptides uniquely identified by orbitrap-based MS, and four are uniquely identified by triple TOF-based MS, and 10 are N-glycopeptides identified by both MS systems using the lectin enrichment method. **D** is the distribution of N-glycopeptides identified by orbitrap-based MS and triple TOF-based MS using hydrazide enrichment. 18 are N-glycopeptides identified by orbitrap-based MS alone, and eight are N-glycopeptides identified by both types of LC-MS/MS instruments with adoption of hydrazide enrichment.

Among those 53 unique N-glycosylated sites, 21 were identified by lectin enrichment alone, eight were uniquely identified by the hydrazide enrichment, and 18 were identified by both enrichment methods using orbitrap-based MS (Figure 2A). Similarly, eight N-glycopeptides were specifically identified by the lectin enrichment protocol, two were specifically identified by the hydrazide chemistry, and six were identified by both enrichment methods using triple TOF-based MS (Figure 2B). In general, 29 N-glycopeptides were uniquely identified by orbitrap-based MS, four were uniquely identified by triple TOF-based MS, and 10 were identified by both MS systems using the lectin enrichment method (Figure 2C). Likewise, 18 N-glycopeptides were identified by orbitrap-based MS alone, and eight were identified by both types of LC-MS/MS instruments with adoption of hydrazide enrichment (Figure 2D).

**Figure 3:**
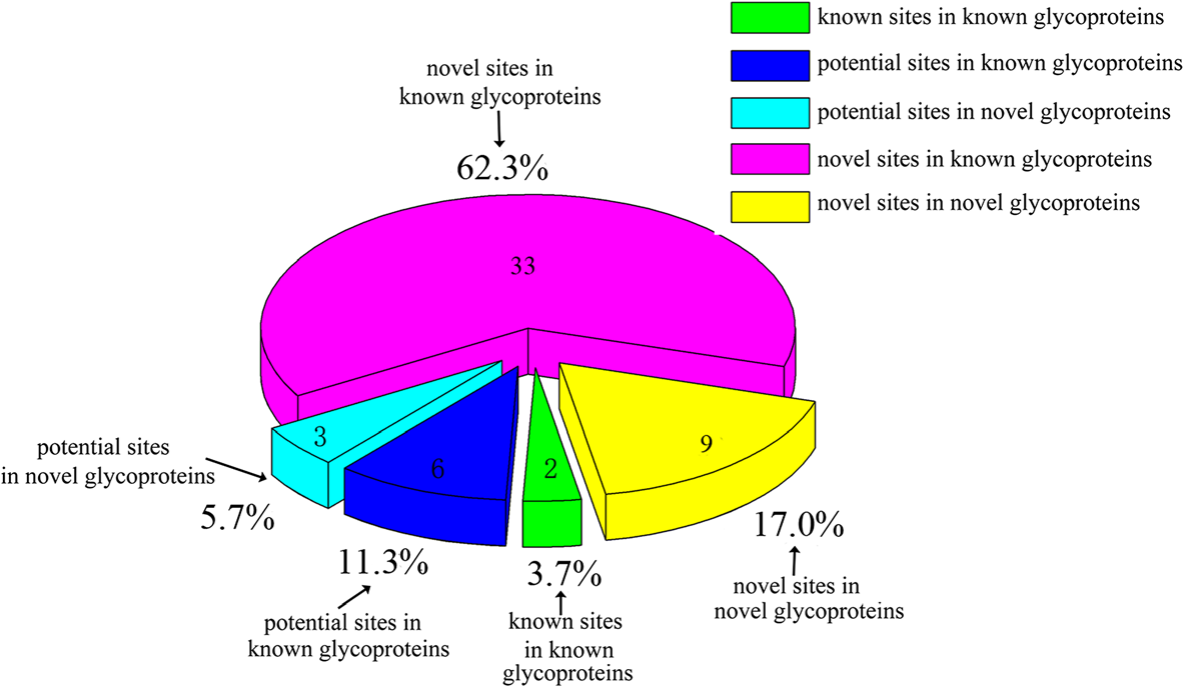
**Distribution of N-glycosylated sites in royal jelly proteins.** “2” is the identified two known sites in known glycoprotein. “6” is potential sites predicted in known glycoprotein, and “3” is potential glycosylation sites identified in novel glycoprotein. “33” is the novel sites identified in known glycoprotein, and “9” is the novel sites identified in novel glycoprotein.

As shown in Figure 3 and Table 2, the distribution of the 53 N-glycosylated sites was subdivided into known and novel proteins. Specifically, only two known sites in known glycoproteins were repeatedly identified in the current study, and six potential sites in known glycoproteins and three potential sites in novel glycoproteins were also identified. The potential sites predicted in the UniProt Database (updated April 2013) were also experimentally confirmed in this study. Thirty-three novel sites were identified in known glycoproteins, and nine novel sites in novel glycoproteins.

**Figure 4:**
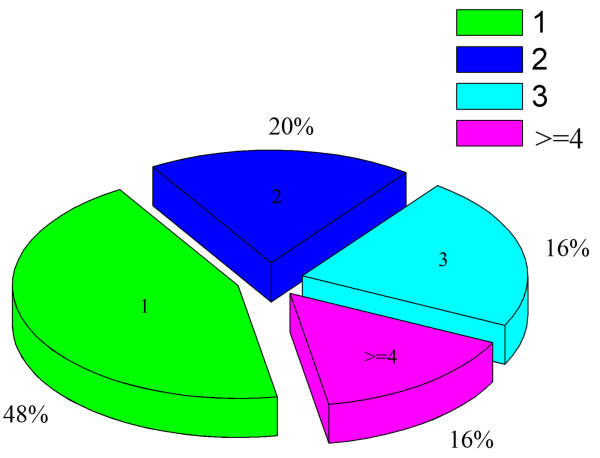
**Distribution of N-glycosylated royal jelly proteins carrying different numbers of modification sites.** “1, 2 and 3” are the N-glycosylated protein carrying 1, 2 and 3 N-linked glycosylation sites, respectvely. “> = 4” is the N-glycosylated protein carried four or more N-glycosylated sites.

Site occupancy analyses showed that approximately 48% of N-glycosylated proteins carrying a single N-linked glycosylated site, 20% contained two sites, 16% retained three sites, and the rest carried four or more N-glycosylated sites (Figure 4).

**Figure 5:**
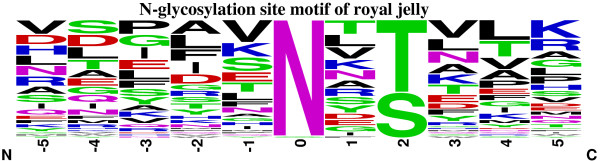
N-glycosylated site motif in royal jelly proteins.

To gain a better understanding the sequence motif of the N-linked glycosylation site in RJ, the surrounding sequences (five amino acids to both termini) of N-glycosylated sites were compared. As shown in Figure 5, about two-thirds were the N-X-T motif and the others were the N-X-S motif in the downstream (positive values) of N-linked modification sites. In other words, the N-linked sequence motif was X-X-N-X-S/T-X in N-glycoproteins of RJ (N = asparagine, X = any amino acid except proline, S/T = serine or threonine).

## Discussion

To gain a new understanding of innate biochemical properties of RJ at the proteome and glycoproteome levels, RJ was analyzed for the identification of novel proteins hidden in RJ and mapped for N-glycosylation sites using the double high LC-MS/MS system (orbitrap and triple TOF) and complementary methods of glycoprotein/glycopeptides enrichment (hydrazide chemistry and lectin). Overall, 13 novel proteins and 42 novel N-glycosylated sites in 25 N-glycosylated proteins were identified.

### Identification of novel RJ proteins

The exploration of novel proteins in RJ is a long-term pursuit for apicultural biologists and biochemical experts. The fast improvement of MS with high resolution, high mass accuracy, and high sequencing speed now allows for in-depth identification of proteins in a comprehensive and unbiased manner in biological samples with high confidence. Compared with previous reports and bioinformatics analysis [1,11,17,28,29], 13 novel proteins were identified in this study. To establish the confidence that the newly identified proteins were real secretory proteins and not contaminated cellular proteins that may have leaked during secretory process of RJ glands, we used two bioinformatics software programs to confirm the origination of the secretory proteins. Proteins predicted as extracellular proteins by PSORT indicate they are putative secretory proteins [30]. To confirm this, SignalP was used to verify the presence of N-terminal secretory signal peptides [31]. This method suggested that all of the 13 novel proteins predicted to be secretory proteins are real protein components of RJ. They are mainly involved in metabolic processes and health promotion activities. This finding is of particular importance for opening new doors to understanding how RJ accomplishes its roles in honeybee biology and in the promotion of human health.

The YELLOW/MRJP is the most important RJ protein family and plays key roles both in honeybee biology and the promotion of human health [9]. The amazing fecundity of the queen (one queen lays 1,500-2,000 eggs a day, more than her body weight [2]) and the exponential speed of larval growth (weight increase by 1,600 times in the first six days of growth [32]) are achieved by a diet of highly-nutritious RJ. MRJPs share a common evolutionary origin with the yellow protein family [33,34]. In particular, *yellow-e3* and *mrjp* genes share the most introns/exons in the same relative positions [33]. The gene expression of *yellow-e3* in the honeybee head and hypopharyngeal glands almost completely coincides with a developmental pattern typical of *mrjp* genes, supporting that *yellow-e3* is the most recent common ancestor of the MRJP families [33,34]. Therefore, the newly identified yellow-e3 precursor in RJ is likely to act in a similar manner to that of the MRJPs, performing multifunctional roles in supplying nutrition and modulating caste determination of the honeybee [34,35]. Noticeably, in previous RJ studies, only MRJP1-5 have been repeatedly identified by a singular proteomics protocol [1,12,17,28]. MRJP6-9 are identified only when special technology is used [8,11]. For example, identification of MRJP8 requires a special digestion method for the proteins [28]. In this study, we not only identified MRJP1-9 in a single study, but we also identified yellow-e3 precursor as a new member of the YELLOW/MRJP family. This indicates that our protocol has a high efficiency in identification of RJ proteins.

RJ provides efficient energetic fuels for the fast development of larvae and the egg-laying queen through the metabolism of sugars, lipids, and proteins [2]. The identification of a high number of proteins related to the metabolism of sugar, lipids, and proteins suggests that the honeybee has an evolutionary strategy of using RJ to fulfill the enormous energy requirement of the fast-developing larvae and the egg-laying queen through these metabolic pathways. Noticeably, five of the 13 novel proteins identified were associated with this category, indicating their biological importance as a source of metabolic fuel for ensuring the normal growth of honeybee larvae. Triacylglycerol lipase breaks down dietary fat, mainly triacylglycerol, to monoacylglycerol, and free fatty acids to supply the energy requirements of living organisms [36]. In addition, enzymes of lysosomal pro-x carboxypeptidase, lysosomal aspartic protease, membrane metalloendopeptidase, and matrix metalloproteinase 14, also participate in the metabolism of protein to produce energy [37,38].

RJ has been well to documented enhance immunity for honeybees and to promote health for humans [2]. Among the 14 RJ proteins related to health promotion activities, six were identified as novel. Dipeptidyl peptidase IV is known to functionally suppress peritoneal dissemination and the progression of ovarian carcinoma, inhibit the malignant phenotype of prostate cancer cells, and promote the human immune system [39,40]. Venom serine protease 34 is part of a defense mechanism against intruding microorganisms and parasites in insects [41-43]. Hymenoptaecin can inhibit the viability of gram-positive and gram-negative bacteria*,* and provides wide-spectrum antibacterial protection for honeybees and humans [44,45]. Venom protease has fibrinogenolytic activity and is a strong antithrombotic agent in snakes [46]. Lysozyme isoform 1 is an important member related to the innate immunity of insects, efficiently protecting larvae from diseases and pests [47]. The hypothetical protein LOC408570 (93% homology with peroxidasin protein of *Harpegnathos saltator*) [48] has functions in phagocytosis and in defense against radioiodinations and oxidation [49].

The newly identified protein cellular repressor of E1A-stimulated genes (protein CREG) might contribute to the promotion of differentiation of honeybee larvae by the enhancement of cell differentiation [50] as MRJP 1 does [51].

### Mapping N-glycosylated sites

By using two complementary enrichment protocols (hydrazide chemistry and lectin resin) and two orbitrap-based and triple TOF-based double high LC-MS/MS systems, we have achieved an in-depth identification of 25 N-glycoproteins that mapped on to 53 sites on RJ proteins. Among these, 42 novel N-linked glycosylation sites were reported in RJ proteins. To the best of our knowledge, this is the most comprehensive assignment of the N-glycosylated sites of RJ.

Capturing the maximum number of glycopeptides is of great importance for the analysis of mapping glycosylated sites [52,53], and is achievable using the complementary enrichment of glycopeptides with techniques such as hydrazide chemistry and lectin based protocols. Hydrazide chemistry can efficiently capture glycoproteins once oxidized by sodium periodate, and is thus extremely useful for the identification of glycopeptides [54]. “Filter aided sample preparation” (FASP) is an N-glycopeptide enrichment protocol that uses a combination of different lectins to efficiently capture glycopeptides [55]. By adopting two different methods based on lectin and hydrazide enrichment, comprehensive glycosylation sites were assigned in RJ, namely 46 by lectin resin and 16 by hydrazide chemistry. Meanwhile, orbitrap-based MS seems to be more robust than Triple TOF-based MS in the identification of glycosylated sites in RJ, and the combined utilization of two different double high LC-MS/MS yielded identification of more number of N-glycosylated sites in RJ. Together, of the 53 N-glycosylation sites assigned in RJ proteins, 42 were mapped as novel. Nine potential N-glycosylation sites predicted by the Uniprot database (updated April 2013) were also verified. In addition, the only two known N-glycosylation sites [16] were repeatedly identified.

It is now known that blocking glycosylation could result in improper or incomplete folding of many polypeptides. These improperly folded polypeptides would not passing ER quality control [56] and would be retained in the ER and eventually degraded [57]. Given that RJ proteins contain 80-90% of MRJPs [9], glycosylation may help MRJPs reach their native conformation to accomplish their biological roles for both honeybees and humans [9]. Glycosylation also allegedly increases the solubility of proteins [58,59]. Therefore, the glycosylated YELLOW/MRJPs suggest their roles in promoting the solubility of YELLOW/MRJPs in RJ to enhance their nutritive efficiency of assimilation [60,61]. Since glycosylated proteins have roles in immunity [62], the weak immunity of the young honeybee larvae (the first 48 h) may be promoted by feeding glycosylated MRJPs to ensure normal development [63]. This is in line with report that glycosylated MRJP 2 can effectively inhibit *Paenibacillus larvae* infection [16].

Glycosylation site occupancy modulates enzymatic activities by the attachment of glycans to peptide backbones [64]. Interestingly, the majority of glycosylated proteins identifed here are enzymes associated with the metabolic pathways of carbohydrates and proteins. For instance, three enzymes, lysosomal alpha-mannosidase, alpha-glucosidase, and glucosylceramidase, are involved in carbohydrate metabolism [65-67]. Four other enzymes, plasma glutamate carboxypeptidase, lysosomal pro-x carboxypeptidase, lysosomal aspartic protease, and membrane metalloendopeptidase, are implicated in the metabolism of proteins [37,38]. The high number of glycosylated proteins related to metabolic processes indicates the production of enough energy through the metabolism of carbohydrates and proteins for queen spawning and larval growth, which may be achieved by modulating the enzymatic efficiency [64].

N-glycosylation modification of proteins has reported to improve the health of living organisms through antibacterial activity [68], antioxidant activity [69], and antihypertension [70]. For instance, glucose oxidase acts as a natural preservative and a bactericide by reducing oxygen to a hydrogen peroxide formation [71]. Venom dipeptidyl peptidase 4 precursor could enhance immune response activity by stimulating the T-cells of mammalia [39,40]. Antithrombin-III, Apolipophorin-III protein precursor, and toll-like receptor 13 all play key roles in promoting the innate immunity of honeybee larvae [11,72-77]. MRJP 1 has potential antitumor effects by stimulating macrophages to release TNF-α [61]. In addition, the glycosylated protein affects cell proliferation and regulates circadian rhythm [78]. Chitinase, as a growth factor, stimulates the proliferation and polarization in *Drosophila*[79]. Protein takeout helps regulation of circadian rhythms and feeding behavior in *Drosophila*[80]. Overall, the glycosylation of these RJ proteins suggests that they may be involved in the above biological roles benefitting both honeybee and humans.

An oligosaccharide unit attached to the polypeptide at the site of occupancy has reported to improve solubility, folding*,* and half-life of the glycoprotein [81]. Most glycosylated RJ glycoproteins (~ 50%) carried a single N-glycosylation site, ~ 20% carried two or three sites, and only a few carried four or five sites. In addition, the identified conservative motif of amino acid sequence of N-glycosylated RJ peptides may have structural and functional importance for RJ proteins in future studies [82,83]. Although the glycan linkages associated with the glycosylation sites demand further investigation, this new catalog of knowledge may prove helpful in elucidating the biological implications of glycosylation for the RJ proteins through synthesizing the glycan to the identified sites. This is possible because N-glycosylation is a conserved process of post-translational modification in a diversity of proteins in eukaryotic organisms [18], and the established N-linked glycosylation system in the *Campylobacter* system could transfer a functional N-linked glycoprotein into *Escherichia coli*[84]*.* This provides promising glycoengineering possibilities for producing modified RJ peptides that could produce a protein with desirable pharmacokinetics and biological activity.

## Conclusions

A total of 13 novel proteins and 42 novel N-linked glycosylation sites in 25 N-glycosylated RJ proteins have been identified here. Of the glycosylated proteins, most were related to metabolic activities and carry multiple N-linked glycosylation sites. This is important for young larvae and the fertile egg-laying queen, since their high metabolic fuel demands may be achieved through the regulation of the enzymatic activities related to the metabolic process. The glycosylated proteins related to the improvement of human health suggest N-glycosylation plays a key role in helping RJ proteins accomplish their biological functions. The large scale assignment of N-glycosylated sites represents a crucial first step toward systematically revealing the functionality of N-glycosylated RJ proteins. In addition, the identification of novel proteins mainly associated with metabolic process and promoting human health significantly extend the proteome coverage of RJ.

## Methods

### Sample preparation

RJ was collected as a pooled samples from 250 queen cell cups from each of five colonies of *Apis mellifera ligustica* at the apiary of the Institute of Apicultural Research, Chinese Academy of Agricultural Science, Beijing. RJ proteins were extracted immediately after harvest according to previously described methods [72]. The resulting pellets were divided into three parts for the following analyses.

### In-solution digestion

The first part of the above protein pellets (1 mg RJ/100 μl buffer) was dissolved in 40 mM of (NH_4_)HCO_3_ (Sigma). The sample was used for in-solution digestion (trypsin, modified sequencing grade, Promega) according to our previous methods [72]. Finally, the peptide-containing solution containing peptides was concentrated using a Speed-Vac system (RVC 2-18, Marin Christ) for MS/MS analysis.

### N-linked glycopeptide enrichment with hydrazide chemistry

The second part of the protein pellet (1 mg RJ/100 μl buffer) was suspended in a coupling buffer [100 mM sodium acetate (Sigma), 150 mM NaCl (Sigma), pH 5.5] and then prepared by enriching the N-linked glycopeptides with hydrazide resin according to the method of Zhang et al. [54]. Briefly, the glycoproteins were oxidized, and these oxidized proteins were captured by hydrazide resin (Bio Rad). The captured glycoproteins were digested overnight by trypsin. Afterwards, the digested glycopeptides were further digested by PNGase F (NEB) to remove the glycans attached to the proteins, and were labeled by H_2_^18^O (Sigma) to confidently assign the N-glycosylation sites. Finally, the collected supernatant was concentrated using a Speed-Vac system for MS/MS analysis.

### N-linked glycopeptide enrichment with lectin

The remaining third of the protein pellets (1 mg RJ/100 μl buffer) was suspended in 8 M of urea in 100 mM of Tris-HCl (pH 8.5) and the mixture was transferred into an Ultracel YM-10 10,000 MWCO centrifugal filter unit (Millipore) and digested by trypsin overnight. Following this, the digested peptides were prepared for enrichment by the N-linked glycopeptides with lectin (mixture with Concanavalin A, wheat germ agglutinin, and RCA_120_ agglutinin) (Sigma) and a second digestion by PNGase F and H_2_^18^O, labeled according to N-Glyco-FASP [85]. Finally, the labeled peptide sample was concentrated using a Speed-Vac system for MS/MS analysis.

### Mass spectrometric analysis

The three peptide samples were analyzed on the Q-Exactive mass spectrometer (Thermo Fisher Scientific) coupled to an Easy-nLC 1000 (Thermo Fisher Scientific) via a nanoelectrospray ion source. Full MS scans were acquired with a resolution of 70,000 at m/z 400 in the orbitrap analyzer. The 20 most intense ions were fragmented by higher energy collisional dissociation (HCD). The HCD fragment ion spectra were acquired in the orbitrap analyzer with a resolution of 17,500 at m/z 400. Reverse phase chromatography was performed with a binary buffer system consisting of buffer A (0.1% formic acid, 2% acetonitrile in water) and buffer B (0.1% formic acid in acetonitrile). The peptides were separated with a flow rate of 350 nl/min in the EASY-nLC 1000 system by the following gradient program: from 3 to 8% buffer B for 5 min, from 8 to 20% buffer B for 55 min, from 20 to 30% buffer B for 10 min, from 30 to 90% buffer B for 5 min, and 90% buffer B for 15 min.

To obtain a comprehensive map of N-glycosylation sites in RJ proteins, the glycopeptide samples were also analyzed by electrospray ionization, quadruple time-of-flight system (Triple TOF 5600, AB SCIEX) coupled with an Eksigent nano liquid chromatography system (Eksigent Technologies). Separation was performed using a self-packed in-house 150 × 0.075 mm 300A pore C18 column, at a flow rate of 330 nl/min. The peptides were eluted with a spectral acquisition speed of 20 MS/MS per second, using the following gradient program: from 5 to 8% buffer B (0.1% formic acid in acetonitrile) for 0.1 min, from 8 to 30% buffer B for 22 min, from 30 to 48% buffer B for 6 min, from 48 to 80% buffer B for 1 min, and 80% buffer B for 5 min.

### Data analysis

Tandem mass spectra were retrieved using Xcalibur (version 2.2, Thermo Fisher Scientific) and AnalystTF (version 1.6, AB SCIEX) software. The MS/MS spectra files were searched against the sequence database (72,672 entries) using in-house PEAKS software (version 6.0, Bioinformatics Solutions Inc.). The database was generated from protein sequences of *Apis* (downloaded April 2012), augmented with sequences from *Sacharomyces cerevisiae* (downloaded April 2012), and a common repository of adventitious proteins (cRAP, from The Global Proteome Machine Organization, downloaded April 2012). The precursor and fragment mass tolerances were set to 50 ppm and 0.05 Da, respectively; tryptic cleavage specificity was set for up to two missed cleavages; carbamidomethyl (C, +57.02) as a fixed modification; and oxidation (M, +15.99) and deamidation (N, +0.98) as the only variable modifications for the RJ sample and oxidation (M, +15.99); deamidation (N, +0.98) and deamidation ^18^O (N, +2.998) for the glycopeptide enriched RJ sample. False discovery rate (FDR) was controlled using a target/decoy database approach for both protein identification and modified peptide identification, applying the cut-off FDR of 0.2%. Protein identification was accepted only if it contained at least two unique peptides. All of the identified glycopeptides and assigned sites were manually checked by applying the cut-off criteria: PEAKS score (-log10P) > 30 and FDR < 0.2%, and the majority of y or b ions could be detected with continuous and strong intensity peaks. To localize protein to the subcellular position, newly identified protein sequences were analyzed by PSORT II Prediction [30] (http://psort.hgc.jp/form2.html). To verify the presence of an N-terminal secretion signal peptide, the SignalP 4.1 Server [31] (http://www.cbs.dtu.dk/services/SignalP/) was also used. The D-cut off for signal-TM networks was set to 0.35. The putative functions of identified proteins and glycoproteins were annotated by searching against the Uniprot database (http://www.uniprot.org/) and grouped on the basis of their molecular behavior and biological process in gene ontology terms. All unique sequences of N-glycopeptides were submitted online to WebLogo [86] in order to extract the N-glycosylated site motif of RJ proteins.

## Availability of supporting data

The data sets supporting the results of this article (Additional file 1: Table S1 and Additional file 2: Figure S1) are included within the article and its additional files.

## Competing interests

The authors declare that they have no competing interests.

## Authors’ contributions

LZ and BH performed experiments related to RJ protein isolation and N-glycoprotein/peptide enrichment, data analysis and the writing manuscript. RLL, XSL, YF and MF performed bioinformatic data analysis. AYN and LHG performed the experiments of liquid chromatography tandem mass spectrometry. JKL participated in the design, coordination and interpretation of the results and the writing manuscript. All authors read and approved the final manuscript.

## Supplementary Material

Additional file 1: Table S1 Identification of Proteins and Peptides in Royal Jelly Proteins. All of the identified proteins are of *Apis mellifera* origin. Accession is the unique number given to mark the entry of a protein in the database of *Apis* (downloaded April 2012, version 4.5 of the honeybee genome). “-10logP” is the score calculated by PEAKS software (version 6.0, Bioinformatics Solutions Inc.). Z is the number of the carrying charge of the peptide. “# Spec” is the number of the spectrum of the peptide. “Start” and “end” correspond to the position of the N-terminal and C-terminal amino acids of the peptide in the protein sequence, respectively. RT is the retention time of the peptide in the mass spectrometry. “ppm” is the deviation value between the experimental mass and the theoretical mass of the peptide. C(+57.02) is the carbamidomethyl modification, M(+15.99) is the oxidation modification, and NQ(+0.98) is the deamidation modification. Click here for file

Additional file 2: Figure S1 Spectra of N-glycosylated peptide in royal jelly proteins. The tandem mass spectrum of the N-glycosylated site is identified in peptide using ^18^O-water labeling. Click here for file
