# Supplementary figures and images for: Comprehensive identification of novel proteins and N-glycosylation sites in royal jelly
Source: BMC Genomics. 2014 Feb 16;15:135. doi: 10.1186/1471-2164-15-135 (PMC3942810; doi:10.1186/1471-2164-15-135)

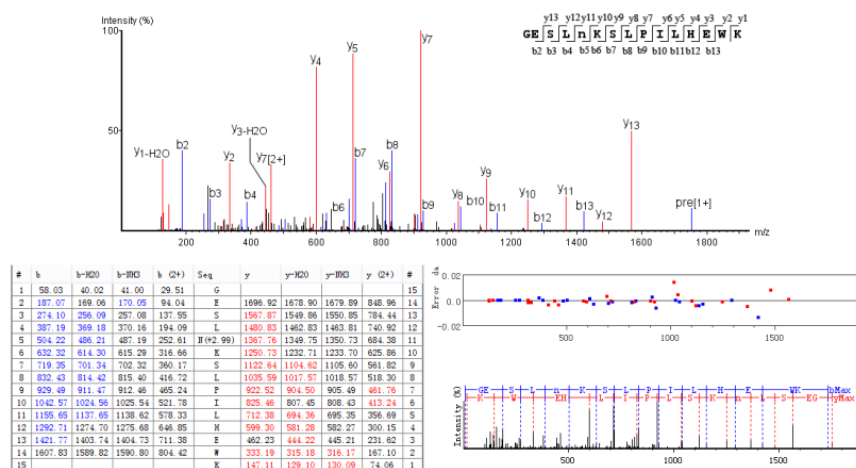

Figure 1

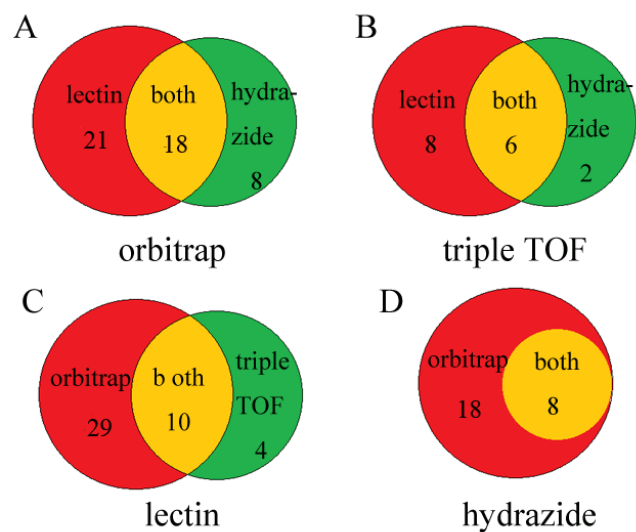

Figure 2

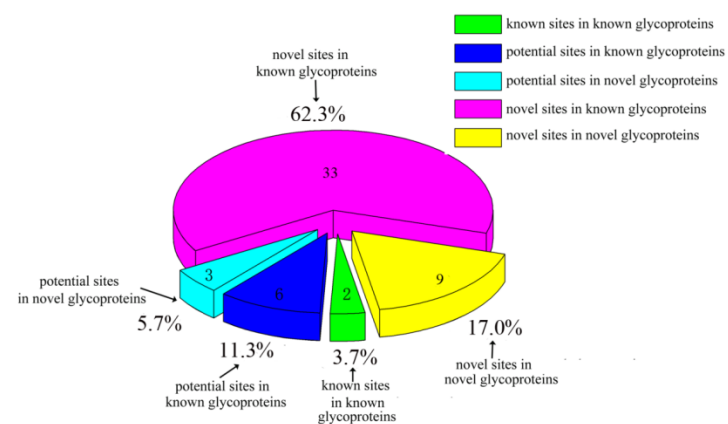

Figure 3

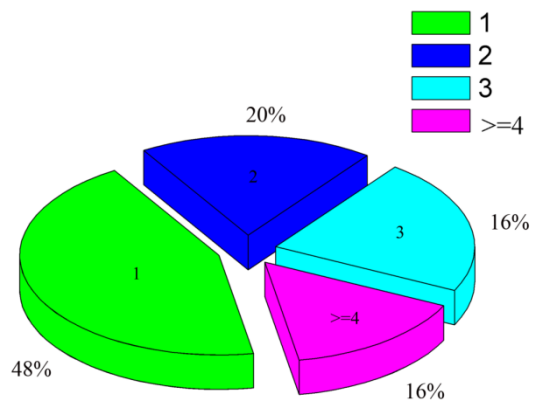

Figure 4

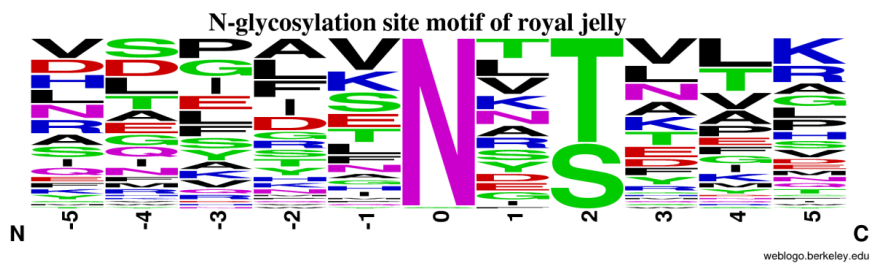

Figure 5

Supplement: Additional file 2: Figure S1 — Spectra of N-glycosylated peptide in royal jelly proteins. The tandem mass spectrum of the N-glycosylated site is identified in peptide using 18O-water labeling. [file 1471-2164-15-135-S2.pdf]
